# Quantifying the demand for hospital care services: a time and motion study

**DOI:** 10.1186/s12913-014-0674-2

**Published:** 2015-01-22

**Authors:** Catharina J van Oostveen, Dirk J Gouma, Piet J Bakker, Dirk T Ubbink

**Affiliations:** Department of Quality Assurance & Process Innovation, Academic Medical Center, P.O box 22700, 1100 DE Amsterdam, The Netherlands; Department of Surgery, Academic Medical Center, Room G4-130, P.O box 22700, 1100 DE Amsterdam, The Netherlands

**Keywords:** Patient characteristics, Demand for hospital care services, Time and motion research, (Multivariable) regression analysis

## Abstract

**Background:**

The actual amount of care hospitalised patients need is unclear. A model to quantify the demand for hospital care services among various clinical specialties would avail healthcare professionals and managers to anticipate the demand and costs for clinical care.

**Methods:**

Three medical specialties in a Dutch university hospital participated in this prospective time and motion study. To include a representative sample of patients admitted to clinical wards, the most common admission diagnoses were selected from the most recent update of the national medical registry (LMR) of ICD-10 admission diagnoses. The investigators recorded the time spent by physicians and nurses on patient care. Also the costs involved in medical and nursing care, (surgical) interventions, and diagnostic procedures as an estimate of the demand for hospital care services per hospitalised patient were calculated and cumulated. Linear regression analysis was applied to determine significant factors including patient and healthcare outcome characteristics.

**Results:**

Fifty patients on the Surgery (19), Pediatrics (17), and Obstetrics & Gynecology (14) wards were monitored during their hospitalization. Characteristics significantly associated with the demand for healthcare were: polypharmacy during hospitalization, complication severity level, and whether a surgical intervention was performed.

**Conclusions:**

A set of predictors of the demand for hospital care services was found applicable to different clinical specialties. These factors can all be identified during hospitalization and be used as a managerial tool to monitor the patients’ demand for hospital care services and to detect trends in time.

## Background

In the upcoming decades the ageing of our population is likely to increase the demand for healthcare services, while more patients will acquire cancer or chronic diseases [1]. This, together with menacing budgetary restraints, will have its impact on hospital resources and may jeopardize the quality, efficiency, and accessibility of patient care [2]. It is therefore important for hospitals to be able to anticipate the upcoming demand for hospital care services, in qualitative and quantitative terms.

Existing instruments to determine the demand for care rely on subjective and clinical observations by nurses, give information about nursing care already given, and help with the staffing of nursing wards [3]. These instruments, however, cannot predict the medical demand for care, and are not solely based on objective measures, such as patient characteristics, e.g. medical diagnosis, age, ASA-classification, etc.

In an earlier study we developed a model for the demand for hospital care services, based on commonly accepted surgical patient characteristics that were readily available from hospital databases [4]. This demand was defined as the costs of (para)medical and nursing resources used by surgical patients during their hospital stay. In this model to predict (trends in) the demand for surgical care, we identified a set of patient characteristics, as well as process and structure variables, i.e.: ASA-classification, number of co-morbidities, polypharmacy during hospitalization, number of complications, surgical specialty, and length of hospital stay (LOS).

To investigate the value of this model in a broader setting, it should be tested among other clinical specialties for its usefulness for hospital managers and policy makers. It may then be used on strategic and tactical levels to determine (trends in) the hospital’s patient population and referral function, to make managerial choices regarding hospital specialization, or to underpin reimbursement negotiations with healthcare insurance companies.

This prospective time and motion study was conducted to find factors related to the demand for hospital care services, based on a previously developed set of factors [4], for some of the most representative clinical patient populations (Surgery, Pediatrics and Obstetrics & Gynecology) in a university hospital.

## Methods

The description and conduct of this time and motion study was done according to the Suggested Time And Motion Procedures (STAMP) checklist [5]. The checklist’s nine main categories of procedures—intervention, empirical setting, research design, task category, observer, subject, data recording, data analysis, and ancillary data—focus on subject identification, randomization, data collection, and data analysis. We used the STAMP checklist to capture and organize the data collection process.

### Setting

The study was undertaken in a tertiary-care university hospital in The Netherlands. Three clinical specialties contributed to the study; five Surgery, four Pediatrics, and three Obstetrics & Gynecology (Ob&Gyn) wards. These subspecialties would introduce a clear variation in age, gender and need for surgery, which were important factors to be taken into account to explain the costs or demand for care.

On each ward approximately 30 nurses, one resident, and several medical specialists contributed during the study. For pediatric and obstetric patients respectively, clinical educationalists, i.e., coaches for hospitalised children, and midwifes were also involved as they contributed a substantial amount of care.

### Design

To estimate the patients’ demand for hospital care we applied a hybrid study design [6], consisting of a so-called “continuous observation time and motion study” among nurses [7], and a paper-based sampling of the activities among physicians. The latter approach was preferred as an equally effective [8], but more reliable method, because during a previous study we had detected considerable under-recording by the physicians when clocking their own activities [4].

### Data collection

In a six-month time frame, we recorded data on hospital features, patient characteristics, diagnostic and surgical procedures, intensive care stay, time spent on patient care by all caregivers involved, and outcome measures to estimate the provided care. Time recordings included direct patient contact (i.e., washing, wound care, communication, ward rounds, etc.) and indirect care (i.e., patient-related telephone calls, planning and administrative activities, inter-professional consultations, multidisciplinary meetings, etc.).

The collected time and resource usage data were used as reference standard for the hospital care needed by the patients in order to test the set of explanatory factors (Table 1). This set was previously developed based on suggestions made by a local expert panel (consisting of head nurses, nursing managers, and physicians), a systematic literature review [9], and results from a previous exploratory study [4]. The factors could be extracted from the patient files and hospital databases and therefore did not require any additional registration effort. Polypharmacy was defined as using 5 or more medications during hospital stay on inpatient wards, being a cutoff point according the Dutch law for Pharmaceutics and commonly used in literature [10].

**Table 1:** **Predictive patient and clinical characteristics investigated**

**Characteristic**	**Range of possible values**
Age	0 to ∞
Ethnicity	0 = Dutch, 1 = Moroccan, 2 = Surinamese, 3 = Other Western, 4 = Other non-Western
Gender	0 = woman or 1 = man
Charlson co-morbidity index (CCI)	0 to 40
Complication severity level	0, 1, 2, 3 or 4
ASA-classification	0, 1, 2, 3 or 4
BMI at admission	NA
Nutritional status (weight loss in past 6 mo.)	NA
Delirium during hospitalization	0 = no or 1 = yes
Pressure ulcer during hospitalization	0 = no decubitus, or grade 1 through 4
Isolated care during hospitalization	0 = no, 1 = barrier or 2 = strict isolation
Survival during hospitalization	0 = yes or 1 = no
Polypharmacy during hospitalization	0 = <5 or 1= >5
Admission type	0 = home or 1 = emergency
Discharge type	0 = home or 1 = other
Readmission within 30 days	0 = no or 1 = yes
Surgical intervention	0 = no or 1 = yes
Medical specialty	Surgery
	Pediatrics
	Obstetrics & Gynecology

*ASA = American Society of Anesthesiologists; BMI = Body Mass Index; NA = not applicable.*

To identify patient characteristics regarding the amount of care needed, we browsed medical and nursing files, either electronic or paper-based. The Charlson co-morbidity index (CCI) was calculated using a calculation sheet [11]. To assess the severity of complications for all patients in the study we used the Clavien-Dindo classification for surgical complications, which is integrated in the national surgical complication registry [12]. Characteristics were collected and checked by two investigators independently during admission and after discharge from the hospital. Missing data were retrieved from medical and nursing files or, if necessary, asked directly from the patients during their hospitalization.

### Patient sample

To include a representative sample of patients admitted to clinical wards of a university hospital, the most common admission diagnoses were selected from the most recent update of the national medical registry (LMR) of ICD-10 admission diagnoses in a whole year. The numbers of patients with the diagnoses to be included were proportionate with the top-20 of admission diagnoses for each specialty (Table 2).

**Table 2:** **Patient samples per medical specialty**

**Specialty**	**Top 12 admission diagnoses ICD 10**	**Planned patient inclusion**	**Included diagnoses**	**Realized patient inclusion**
Surgery	**Diseases of the genitourinary system**	3	Donor nephrectomy	1
			Hyperplasia of prostate	1
	**Diseases of the digestive system**	1	Perforation of bile duct	1
	**Neoplasms (Orthopedics, Urology and GI-surgery)**	7	Malignant neoplasm prostate	2
			Malignant neoplasm extrahepatic bile ducts	2
			Malignant neoplasm esophagus	1
			Malignant neoplasm pancreas	1
			Secondary malignant neoplasm liver	1
			Malignant neoplasm of thyroid gland	1
			Malignant neoplasm femur	1
	**Diseases of the circulatory system**	3	Claudication Intermittent, Fontaine stage III	1
			Claudication Intermittent, Fontaine stage IV	1
			Aortic aneurysm	1
	**Injury, poisoning and certain other consequences of external causes**	2	Fracture femur	1
	**Diseases of the musculoskeletal system and connective tissue**	2	Osteoarthrosis	1
			Infection following a procedure after fracture of forearm	1
			Pyogenic arthritis	1
**Total**		**18 (100%)**		**19 (106%)**
Pediatrics	**Diseases of the respiratory system**	3	Pneumonia	1
			Emphysema	1
	**Diseases of the musculoskeletal system and connective tissue**	2	Other disorders of bone development and growth	1
			Pectus Excavatum	1
	**Neoplasms**	4	Benign neoplasm tonsil	1
			Neuroblastoma	1
			Malignant neoplasm femur	1
			Secondary malign. neoplasm bone and bone marrow	1
			Lymphoid leukemia	1
	**Diseases of the ear and mastoid process**	1		0
	**Injury, poisoning and certain other consequences of external causes**	2	Concussion	1
	**Diseases of the digestive system**	5	Anal abscess	1
			Constipation	1
			Fistula of urethra to small intestine	1
			Esophageal obstruction	1
			Cholestasis	1
			Acute appendicitis	1
	**Congenital malformations, deformations and chromosomal abnormalities**	1	Hirschsprung’s disease	1
**Total**		**18 (100%)**		**17 (94%)**
Obstetrics & Gynecology	**Neoplasms**	7	Malignant neoplasm of vulva	1
			Malignant neoplasm of cervix	1
			Malignant neoplasm of ovary	2
			Secondary malignant neoplasm of perineum	1
			Benign neoplasm of ovary	1
			Leiomyoma of uterus	1
	**Diseases of the genitourinary system**	3	Prolapse of vagina wall	1
	**Pregnancy, childbirth and the puerperium**	9	Maternal care for (suspected) damage to fetus by drugs	1
			Maternal care for other known or suspected fetal problems	1
			Preterm labor without delivery	1
			Vaginal delivery following previous caesarean section	1
			Single spontaneous delivery	1
			Labor and delivery complicated by fetal stress	1
**Total**		**19 (100%)**		**14 (74%)**
***Overall total***		***55 (100%)***		***50 (91%)***

### Study conduct

#### Time and motion

All contributing caregivers were informed about the study purpose and the use of the PDAs for the time recordings through instruction briefings, a study website, and a YouTube clip [13]. We continuously monitored the patients’ care process during the whole admission period.

**Figure 1:**
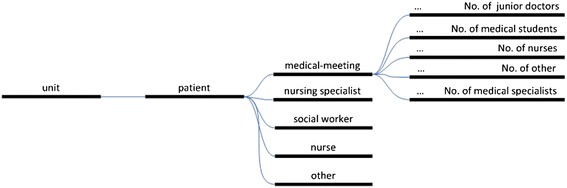
**Menu structure of personal digital assistants.**

For the continuous time and motion recordings by nurses, Personal Digital Assistants (PDAs) (PalmOne Tungsten E2; Palm Inc., Sunnyvale, CA, USA) were used. These PDAs were equipped with a dedicated software program (I-V-O: Web development, scripting, hosting & consultancy, Alkmaar, The Netherlands). This enabled the recording of the date, duration of direct and indirect time spent per patient, types of caregivers involved, and wards and patients involved (Figure 1). The different activities were not discerned in the recordings. Many of the nurses were familiar with the time clocking procedure as they had participated in the previous study as well. The researchers distributed the PDAs and supported the nurses’ recording activities. Two PDAs were placed at the patients’ bedside, one of which was placed in a charger. The nurse taking care of an included patient was to carry one of the PDAs and use it as a stopwatch to clock the time (s)he spent on that particular patient and hand it over to the next colleague at the end of their shift, seven days a week for 24 hours a day, until the patient was discharged from the hospital. If another nurse would visit the patient, (s)he could use the other PDA at the patients’ bedside. Data collected in the PDAs were downloaded into the database after the patient’s discharge.

Physicians were instructed to record the time they spent on the included patients on a daily documentation form, distributed by the coordinator. This form was used to record the date, patient study number, and minimum and maximum estimates of the direct and indirect time spent per patient. The researchers contacted the physicians on average every other day by phone, e-mail, or face to face, to fill out the documentation form if not yet done so.

To avoid measurement errors, nurses and physicians were allowed to measure the time spent for a maximum of 3 patients simultaneously. Time data recorded by nurses were checked and analyzed after the patients’ discharge for outliers or missing values. Patients were excluded from the analysis if the time recordings covered less than 50% of their hospital stay. To check the reliability of the data we frequently monitored and asked the caregivers involved regarding their recording behavior.

To evaluate whether the patients felt their demand for care was met during their admission, patient satisfaction with physicians’ and nurses’ attention and knowledge was asked by means of a short questionnaire, on their day of discharge. Additionally, to account for a possible influence of the availability of personnel resources on the amount of care given, we also assessed the proportion of rejected patients on the contributing wards during the study period. Reasons for rejection were maximum bed occupation and unavailability of necessary resources.

#### Time and costs as measure of the demand for hospital care services

The demand for hospital care services was based on the time spent by the caregivers on the one hand, and costs of diagnostic and therapeutic interventions on the other. To produce a single measure for the demand for care, we also converted these time data into costs and added them to the intervention costs.

Standard 2012 salary costs of the various caregivers involved were used for day, weekend, and night shifts, according to the national manual for research on hospital costs [14]. Costs of surgical interventions were based on the gross time spent on the surgical procedure and the associated salary costs of the caregivers present. For costs of diagnostic and therapeutic procedures, ICU and recovery stays, standard tariffs were extracted from the hospital ledger. These costs represent material and overhead costs for procedures and additional personnel costs for ICU and recovery stays. The overhead costs of indirectly involved personnel (for example managers, administrative personnel, patient transport officers) were not taken into account.

The sum of the costs for the time spent by caregivers and the costs for diagnostic and therapeutic procedures, ICU and recovery stays represent direct patient costs and were taken as a measure for the demand for hospital care services for each patient’s hospital stay. The contributing parts of these costs were presented in stacked bar graphs as percentages of the total costs, normalized at 100%. Subsequently, these total costs were used as the dependent variable in the regression analyses.

### Data analysis

Data were imported and analyzed in the Statistical Package for the Social Sciences v. 20 (IBM SPSS Inc., Armonk, NY, USA). Categorical data are presented as proportions. Continuous variables are summarized as means with standard deviations, or medians if not normally distributed. A multiple backward linear regression analysis was done with characteristics that were associated (p < 0.20) with the costs of the demand of care in a univariable analysis. B-values, as a measure of the association with the costs of the demand for care, were calculated with their 95% confidence intervals. Log-transformation of the dependent variable “total costs of demand for care” was performed because of its non-normal distribution.

### Ethical issues

Our local medical ethics review board (Academic Medical Center, Amsterdam, The Netherlands) approved the study but waived the need for written informed consent, as the study had no effect on the patients’ treatment or psychological wellbeing.

## Results

Within the study’s time frame from July to January 2013, we included 78 consecutive patients, both elective and emergency admissions. After data validation, 50 valid patient cases remained for analysis (Table 2). Characteristics of included patients are summarized in Table 3. The patients were evenly divided among the medical specialties. Median LOS was 5 days (range: 1–51). Almost 30% of the patients underwent a surgical intervention and more than 30% were re-admitted within 30 days.

**Table 3:** **Univariable and multivariable linear regression analysis of possible predictive characteristics**

				**Univariable**				**Multivariable**			
**Characteristic**	**N (%)**	**Mean (SD)**	**Range**	**Β**	**β**	**CI**	**P**	**Β**	**β**	**CI**	**P**
Age		43 (28.90)	1-92	0.004	0.281	0.000-0.008	0.048				
Ethnicity											
Dutch	39 (78)			RC	-	-	-				
Moroccan	2 (4)			−0.237	−0.112	−0.861-0.387	0.449				
Surinamese	2 (4)			−0.068	−0.032	−0.692-0.556	0.827				
Other Western	3 (6)			−0.020	−0.012	−0.536-0.496	0.938				
Other non-Western	4 (8)			−0.292	−0.191	−0.744-0.160	0.200				
Gender (males)	19 (38)			0.047	0.055	−0.201-0.295	0.704				
Charlson co-morbidity index											
No comorbidity	30 (60)			RC	-	-	-				
1	4 (8)			0.093	0.061	−0.344-0.530	0.669				
2	5 (10)			0.338	0.244	−0.059-0.734	0.093				
3	5 (10)			0.492	0.355	0.095-0.888	0.016				
4	1 (2)			0.471	0.159	−0.363-1.305	0.261				
5	1 (2)			0.081	0.027	−0.753-0.915	0.845				
6	4 (8)			0.169	0.110	−0.268-0.606	0.440				
Complication severity level											
No complication	46 (92)			RC	-	-	-	RC	-	-	-
1	3 (6)			0.649	0.371	0.203-1,094	0.005	0.439	0.251	0.077-0.801	0.018
2	1 (2)			1,020	0.344	0.265-1.776	0.009	0.811	0.273	0.208-1.414	0.010
3	0 (0)			-	-	-	-	-	-	-	-
ASA-class											
No ASA-class	12 (24)										
1	19 (38)			RC	-	-	-				
2	14 (28)			0.479	0.571	0.226-0.732	<0.001				
3	5 (10)			0.155	0.130	−0.205-0.516	0.388				
BMI at admission		23.16 (6.61)	13.19-42.06	0.015	0.008	−0.002-0.032	0.086				
Nutritional status		0.93 (0.37)	0.00-10.00	0.053	0.274	−0.007-0.112	0.083				
Delirium during hospitalization	0 (0)										
Pressure ulcer acquired during hospitalization	0 (0)										
Isolation											
barrier	1 (2)										
strict isolation	0										
Survival	50 (100)										
Polypharmacy		7.80 (4.90)	0-21	0.309	0.341	0.061-0.556	0.015	0.213	0.235	0.027-0.399	0.026
Admission type											
home	47 (94)										
emergency	3 (6)										
Discharge type											
Home	48 (96)										
Other	2 (4)										
Surgical Intervention performed	14 (28)			0.537	0.581	0.318-0.755	<0.001	0.461	0.499	0.271-0.651	<0.001
Medical specialty											
Surgery	19 (38)			RC	-	-	-				
Pediatrics	17 (34)			−0.340	−0.389	−0.608--0.073	0.014				
Obstetrics & Gynecology	14 (28)			−0.046	−0.050	−0.328-0.236	0.745				

*ASA = American Society of Anesthesiologists; BMI = Body Mass Index.*

Median time spent on the care of patients by physicians and nurses per admission was 15.5 hours and varied from 3.3 hours for a pediatric patient with lymphoid leukemia to almost 4 days for a patient with complications from a malignant neoplasm of the femur. Total time spent by nurses and physicians was 40 days and 6 hours, the majority of which (71%) was spent by nurses. The time spent by physicians did not include the time involved in surgical interventions. Surgeons spent almost half an hour for a tonsillectomy up to almost 12 hours for an extended hemi-pelvectomy with reconstruction in the operating room.

**Figure 2:**
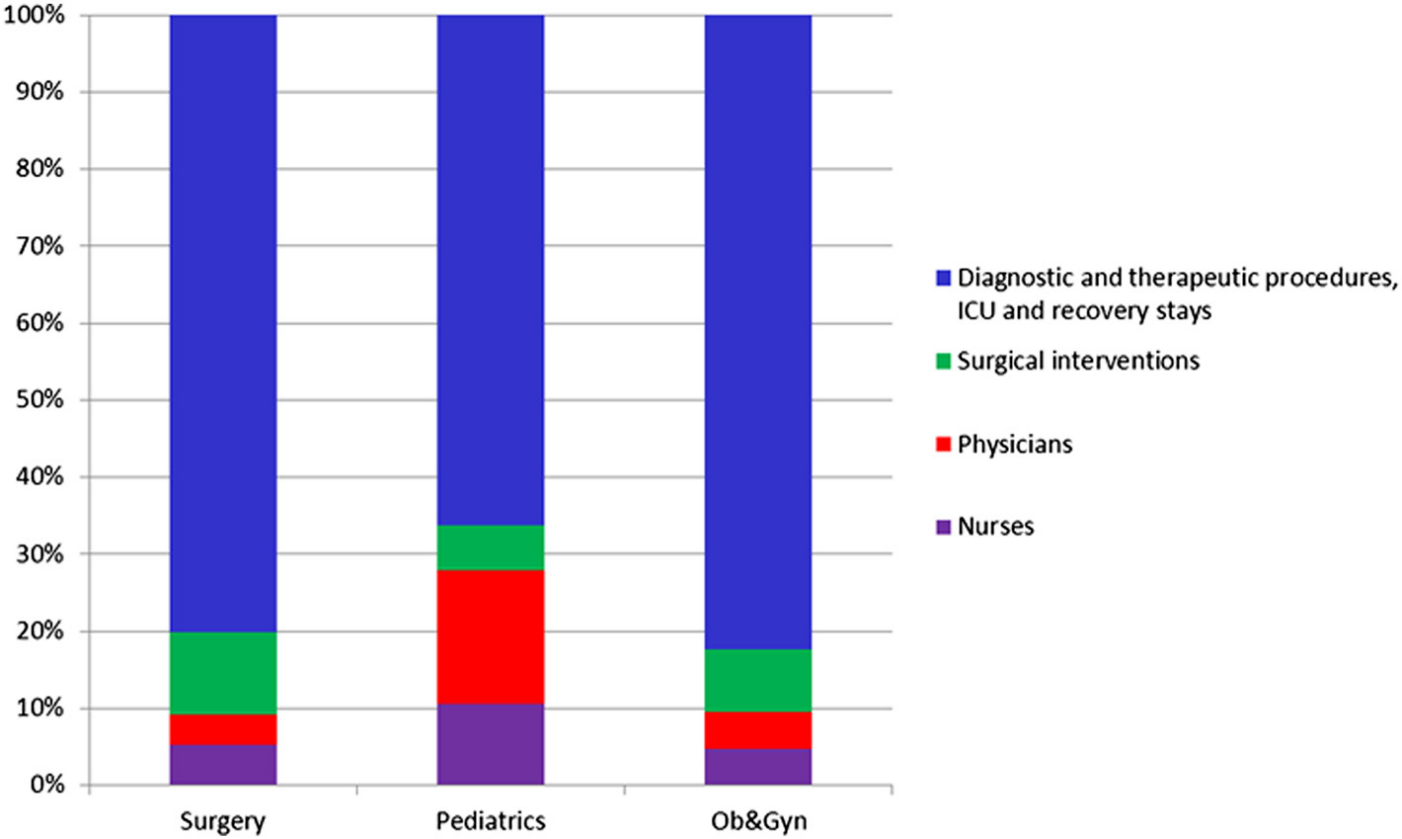
**Cost distribution of the demand for care per specialty. ** Ob&Gyn = Obstetrics and Gynecology; ICU = intensive care unit.

Sum total costs of the demand for care per specialty varied from €127.000 for pediatric patients, €70.500 for Ob&Gyn patients to €206.500 for surgical patients. Surgical and diagnostic interventions contributed most to these overall costs (Figure 2). In the univariable analysis, age, CCI, complication severity level, ASA-class, BMI, nutritional status, polypharmacy (defined as the use of 5 or more medications during hospitalization), whether a surgical intervention was performed and specialty were associated with the costs of demand for care (Table 3), as opposed to gender, ethnicity, and readmission within 30 days. Delirium and isolation during hospitalization, pressure ulcers, admission and discharge type, and mortality did not contribute significantly.

All significant characteristics in the univariable analysis were entered in the multivariable regression analysis (Table 3). Independent factors associated with the total costs of the demand for care were: surgical intervention performed, polypharmacy during hospitalization, and complication severity level, but not age, ASA-class, BMI, nutritional status, surgical intervention, or specialty. This model explained 55% of the variance in the demand for care in terms of costs. Whether or not patients underwent surgery explained 33.7% of this variance. Undergoing surgery led to a cost increase of 215% (95% CI 65% to 348%; p <0.001). When shifting from no complications to complication severity level 1, total costs increased significantly with 78% (95% CI 19% to 532%; p = 0.018), and with 87% (95% CI 61% to 2494%; p <0.001) when moving from no complications to complication severity level 2. Polypharmacy was responsible for a 72% (95% CI 0.6% to 150%; p = 0.026) increase in costs.

Approximately 95% of the patients were satisfied with the treatment they received. Nurses’ and physicians’ competences and the attention patients received from them were also judged as satisfactory. Only 2% of the patients were “moderately satisfied” with the attention from physicians and nurses. Hence, the care provided seemed to be in agreement with the patients’ demand for care (Table 4).

**Table 4:** **Results of the questionnaire evaluating provided care**

**Question**	**Answer options**	**N (%)**
Satisfied with treatment	Very satisfied	33 (66)
	Satisfied	14 (28)
	Fairly satisfied	3 (6)
	Moderately satisfied	0 (0)
	Dissatisfied	0 (0)
Satisfied with nurse attention	Very satisfied	30 (60)
	Satisfied	18 (36)
	Fairly satisfied	0 (0)
	Moderately satisfied	1 (2)
	Dissatisfied	1 (2)
Satisfied with nurse competence	Very satisfied	34 (68)
	Satisfied	14 (28)
	Fairly satisfied	2 (4)
	Moderately satisfied	0 (0)
	Dissatisfied	0 (0)
Satisfied with physician attention	Very satisfied	32 (64)
	Satisfied	13 (26)
	Fairly satisfied	4 (8)
	Moderately satisfied	1 (2)
	Dissatisfied	0 (0)
Satisfied with physician competence	Very satisfied	41 (82)
	Satisfied	8 (16)
	Fairly satisfied	1 (2)
	Moderately satisfied	0 (0)
	Dissatisfied	0 (0)

Rejection rates as a proportion of the total number of admitted patients during the study period varied from 1.2% for surgical wards to 2.8% for pediatric wards. Hence, we assumed that the availability of resources had no meaningful influence on the demand for hospital care services.

Based on our random checks of the completeness of data recordings physicians appeared to record between 80 to 100% of their activities.

## Discussion

This time and motion study shows that the previously found set of independent factors explaining the surgical patients’ demand for hospital care services are also applicable to various other clinical specialties, as the specialty per se was not found to be an independent correlate. This set comprises patient characteristics that become apparent during hospitalization, i.e., polypharmacy during hospitalization, complication severity level, and whether a surgical intervention was performed. Although the need for surgery may be clear in advance, only 24% of our study population underwent surgery while the surgical intervention was not the reason for admission for most of these patients. This implies that the demand for hospital care can be assessed during and after the patient’s hospitalization rather than predicted in advance. This makes the set of characteristics useful as a managerial tool to retrospectively assess the (trends in the) demand for care on various wards within a clinical specialty. For this purpose, these characteristics should be recorded and readily available in hospital databases.

The effect on costs of polypharmacy during hospitalization as found here is larger than in two previous studies [4,15], probably due to the more expensive medical treatments included in this study, e.g. chemotherapy and the fact that the effect found represent 5 or more medications instead of one. This may be exemplary for the university hospital in which this study was conducted.

Complications occurring during hospitalization had a large effect on costs, as opposed to our previous study. In the present study the complication severity level was used rather than the number of complications, as chosen in our previous study [4]. By doing so, fewer complications were taken into account as patients could have more complications within the same severity level. Hence, the incidence of complications, especially in the higher levels, was quite low. Age was not found as an independent factor, which corresponds with the findings in other studies [4,15,16].

The weighted Charlson measure for co-morbidity (CCI) [11], was not found to be an independent characteristic. This is in contrast with other studies [17,18], although these did not find large effects on care costs. This discrepancy could be caused by the fact that these studies used more homogeneous patient groups in protocol-based care pathways, i.e. after hip replacement [18], or with heart-failure [17]. Furthermore, caregivers may be reluctant to treat patients with a high co-morbidity with invasive therapies, e.g. surgery, which obviously increases hospital costs. Finally, the fact that the CCI was validated in adult patients makes its application in children doubtful. We therefore cannot state that comorbidity, particularly in children, is not a relevant factor. Only recently a comorbidity measure has become available for pediatric patients [19].

The influence of several possible factors, for example, delirium and isolation during hospitalization, pressure ulcers, admission and discharge type, and mortality, could not be appreciated because of their low incidences. Hence, these are not likely to substantially influence the total costs of care for this patient population.

### Strengths and limitations

One of the strengths of this study is that we were able to reliably record the time spent by physicians. Earlier, we found an under-recording by physicians [4]. We therefore adapted our data collection method for this discipline, resulting in completeness of the data recorded. Thus, a more exact estimation of the factors associated the demand for hospital care (services) by physicians. Also, the cost estimation of diagnostic and therapeutic procedures, including surgical interventions, could be performed more precisely by using cost prices including overhead and material costs instead of mere personnel costs. On the other hand, it was not feasible to take into account the costs for overhead, patient transport, and medication on the nursing wards, and to correct for the additional charges for physicians during weekends, evenings and nights. This might explain why the costs for diagnostic and therapeutic procedures contributed most to the overall cost estimation of the demand for hospital care.

Limitations of this study are, in the first place, the observed high re-admission rate, which might seem a sign of a poor outcome. However, a considerable part of our patient population was treated for neoplasms by means of radiotherapy or chemotherapy, which explains the frequent re-admissions. Furthermore, patient satisfaction was high and rejection rates were low for every specialty involved. This suggests that the demand for care was measured rather than the care that could be offered, which could have been limited by available resources and personnel.

Second, we started this study as a multicenter validation study to obtain a larger set of representative patients from various clinical specialties. Unfortunately, it turned out to be impossible to collect reliable data in other hospitals because of logistic problems, motivational problems and financial issues in times of budget restraints. As a result, the study population was limited to our own hospital. This limited the number of factors to be tested and the precision of our results. Also, no organizational factors, which may differ among hospitals, could be included. However, in our univariable regression analysis only three factors were found to significantly influence the demand for hospital care services. Therefore, the number of included patients was sufficient to test all three factors in the multivariable analysis, which are in keeping with the previously found set [4].

Third, we were not able to include all major clinical specialties, like internal medicine. Therefore, the outcomes of our study are applicable to a large proportion, but not all of the clinical hospital population. Furthermore, patient inclusion in this study was based on our national registration of clinical admission diagnoses. The sample of diagnoses we chose was representative for university medical centers in the Netherlands. Here, tertiary-referral university hospitals mainly provide academic patient care, while simple, short-stay admissions mostly occur in affiliated general hospitals. This may imply that the patient mix investigated here may differ from patients treated in university centers in other countries.

Fourth, in obstetric patients we started measuring the care given to one, but after delivery to two (or even more) subjects. As patient characteristics of only the mother were included, this could have had impact on our study outcomes. However, this is the actual way how mother & child care is organized in the Netherlands. Thus, managers and policymakers should take this into account when interpreting these findings.

## Conclusion

To assess the demand for hospital care as given by various clinical specialties, a set of patient characteristics could be defined, based on a previous model developed in surgical patients. This set of factors comprise polypharmacy during hospitalization, complication severity level, and whether a surgical intervention was performed. These can be obtained during hospitalization and can be used as a managerial tool to monitor the amount of care on a more aggregate level on wards to detect trends in time as to patients’ demand for hospital care services. A practical implication could be the integration of these factors in a management information dashboard. This information allows managers and policy makers on strategic and tactic levels in evaluating, planning and budgeting on real data, i.e. to make choices regarding hospital specialization, to substantiate the hospitals (top)referral character and to support negotiations with healthcare insurers. On the other hand, assessment of the demand for hospital care as a predictive tool before patient admission remains difficult.
